# O’nyong-nyong virus adaptive mutations in non-structural protein 1 and 3 enhance RNA replication and overcome FHL1 requirement

**DOI:** 10.1080/22221751.2026.2721742

**Published:** 2026-08-19

**Authors:** Tessy A.H. Hick, Karsten Cirksena, Magnus Evander, Gisa Gerold

**Affiliations:** a Department of Clinical Microbiology, Umeå University, Umeå, Sweden; b Umeå Centre for Microbial Research (UCMR), Umeå University, Umeå, Sweden; c Department of Medical and Translational Biology, Umeå University, Umeå, Sweden; d Research Center for Emerging Infections and Zoonoses, University of Veterinary Medicine Hannover, Hannover, Germany; e Cellular Proteome Research, Helmholtz Centre for Infection Research Braunschweig, Braunschweig, Germany; f Department of Hygiene, Microbiology and Public Health, Institute of Virology, Medical University of Innsbruck, Innsbruck, Austria

**Keywords:** Alphavirus, o’nyong-nyong virus, FHL1, adaptive mutations, non-structural proteins

## Abstract

Arthritogenic alphaviruses, like o’nyong-nyong virus (ONNV), cause debilitating musculoskeletal diseases and are geographically expanding. To predict their emergence, we seek to better understand evolutionary mechanisms that enable changes in virus tropism. Here, we identify adaptive mutations in the ONNV non-structural proteins (nsPs) that arose during cellular serial passaging and enabled ONNV to infect non-permissive Lunet cells. Using shotgun proteomics, we show that this human hepatoma cell line lacks the four-and-a-half-LIM domain protein 1 (FHL1), an essential host factor in ONNV RNA replication. Individual single nucleotide mutations in the nsP1 ring-aperture membrane-binding and oligomerization domain, the nsP3 macrodomain, and the nsP3 opal stop codon overcome FHL1 deficiency in Lunet cells by enhanced RNA replication. These findings demonstrate how subtle genomic changes in nsPs can profoundly influence alphavirus replication and tropism.

## Introduction

O’nyong-nyong virus (ONNV) is a neglected, mosquito-borne alphavirus (family *Togaviridae*) causing re-emerging outbreaks of febrile illness in sub-Saharan Africa [1]. Infection is characterized by rash, fever, and severe polyarthralgia, which is typically self-limiting, although prolonged duration of the symptoms has been reported [1–3]. The symptoms resemble those of chikungunya virus (CHIKV), a phylogenetically closely related alphavirus of global concern. Given ONNV’s travel-associated geographic dissemination [4,5], climate-driven expansion of its *Anopheles* mosquito vector [6], and potential transmission by other mosquito vectors [7,8], ONNV represents a potential emerging health threat beyond the African continent.

The re-emergence of RNA viruses, such as ONNV, is closely linked to their genome plasticity. Most RNA viruses lack proofreading activity in their RNA-dependent RNA polymerase (RdRp), which makes them error-prone and constantly results in novel variants [9]. Nevertheless, mosquito-borne RNA viruses show a high degree of consensus genome stability, maintained through their alternating replication in mosquito and vertebrate hosts [10–13]. The dual host cycling constrains evolution to mutants that gain a fitness advantage in one host without negatively affecting infection in the other, e.g. general advantages in virion stability [14], or RNA replication [15,16].

The alphavirus positive-strand RNA genome contains two open reading frames encoding non-structural and structural proteins [17]. The structural proteins, i.e. capsid and envelope glycoproteins, constitute the spherical virion [18,19], whereas the non-structural proteins (nsPs) assemble into the viral RNA replication complex. This complex associates with the plasma membrane and remodels it into invaginations called spherules [20]. The complex is anchored by nsP1 which forms a ring structure that serves as capping pore and assembly hub for the three other nsPs [21,22]. NsP2 and nsP3 are located on the cytosolic side of the ring, where the nsP2 RNA helicase has nucleoside triphosphatase and RNA triphosphatase activities [23–26]. nsP3 can assemble into tubular scaffolds that interact with multiple host proteins essential in the RNA replication [27–29]. The nsP4 RdRp is situated in the central pore and synthesizes viral RNA in a well-coordinated manner [20–22]. Although our understanding of the replication complex has advanced through structural characterization of the late replication complex, the detailed architecture of the initial replication complex and its interactions with host proteins has yet to be elucidated [30]. In the initial composition of the replication complex (nsP123 precursor and nsP4), it synthesizes negative strand RNA. This synthesis transitions to genomic RNA synthesis by nsP2 proteolytic cleavage of the nsP123 precursor into nsP1 + nsP23. Lastly, cleavage of nsP2 + nsP3 results in subgenomic RNA synthesis [19,31–33]. The molecular mechanisms and the conformational rearrangements that drive this transition remain poorly understood.

Alphavirus RNA replication is greatly dependent on host factors that are required for the formation of RNA replication complexes. Over the last years, several host dependency factors have been identified. Some are conserved across alphaviruses (e.g. CD81) [34], some are exploited by either New World (e.g. FXR) or Old World alphaviruses (e.g. G3BP) [35], and some are unique to specific alphaviruses, e.g. four-and-a-half-LIM domain protein 1 (FHL1) for CHIKV and ONNV [36–38]. FHL1 increases CHIKV and ONNV infectivity by its binding to nsP3 hypervariable domain early in replication [38]. Although FHL1 is an essential host factor for the majority of ONNV and CHIKV strains, several strains can replicate independent of FHL1 [36–38]. Notably, FHL1 deficient mice show milder disease symptoms and foot swelling upon CHIKV infection [37]. As the abundance of FHL1 in muscle cells correlates with the tissue tropism of ONNV and CHIKV, it likely contributes to the characteristic arthritic disease [36].

In this study, we discovered evolutionary single nucleotide mutations in the non-structural genes that allowed an ONNV infectious clone (derived from a clinical serum isolate) [39,40] to overcome FHL1 deficiency in a non-permissive human liver cell line, Lunet cells. Various mutants established spontaneously during serial passages in mammalian and mosquito cell lines and demonstrated efficient infection in Lunet cells. We examined four mutations in nsP1 and nsP3 in detail, which demonstrated enhancement of RNA replication. Each of the four mutations rendered Lunet cells ONNV permissive and increased virus proliferation in other cells. Our study provides insights into the genomic plasticity of ONNV, which influences RNA replication and virus tropism.

## Materials and methods

### Cells and viruses

Lunet (Huh 7 derived) [41], Huh7.5 [42], Lunet human CD81 [34], HEK293, HEK293 ΔFHL1 [36], Vero B4 [43], A549 [44], and Human Fibroblast [45] cells were cultured at 37°C and 5% CO_2_ in Dulbecco’s modified Eagle medium (DMEM, Sigma) supplemented with 10% fetal bovine serum (FBS, Gibco), 1% penicillin-streptomycin (P/S, Sigma-Aldrich), 1% nonessential amino acids (NEAA, Gibco), and 1% L-glutamine (Gibco). C6/36 cells were cultured at 28°C in Leibovitz's L-15 medium (VWR) supplemented with 10% FBS, 1% P/S, 1% NEAA, and 2% tryptose phosphate broth (ThermoFisher). MSQ43 cells (BEI Resources contributed by Shirley Luckhart and Imogene Schneider) were cultured at 28°C in Modified Eagle’s medium with Earle’s salts (VWR) supplemented with 5% FBS, 1% P/S, 2% NEAA, 2% L-glutamine, 1% glucose (ThermoFisher), and 1% vitamin solution (ThermoFisher).

Genetically modified cells were generated using lentivirus vector transduction. HEK293 and Lunet cells were generated to stably express the Tet3G promoter and the Tet3G-controlled ZsGreen1 reporter protein, respectively, as described in [46]. In brief, the plasmids pLVX-Tet3G and pLVX-Tre3G-ZsGreen1 (Takara Bio) were transduced into cells using lentivirus vectors and selected using 800 µg/ml geneticin (G418, Gibco) and 0.5 µg/ml puromycin (Gibco), respectively. The stable cell lines were maintained in culture media with 10% Tet system-approved FBS (Gibco) and either 800 µg/ml G418 or 0.5 µg/ml puromycin. To generate FHL1-expressing Lunet cells, Lunet and Lunet CD81 cells were transduced with pLVX-FHL1-ZsGreen1 (kind gift of Prof. Ali Amara, Université de Paris, France) lentivirus vectors as described in [36].

Virus stocks of ONNV RRC013 SG mCherry (referred to as ONNV WT) [39], VEEV TC-83 SG eGFP [47,48], and CHIKV 37997 (West African) E2 mCherry [49] were obtained by transfection (Lipofectamine3000, Invitrogen) of *in vitro* transcribed (mMESSAGE mMACHINE SP6 Transcription Kit, ThermoFisher) infectious RNA in Vero cells. The ONNV-CHIKV chimaeras were received from Prof. Andres Merits, University of Tartu, Estonia. In short, the chimeric viruses were constructed using plasmids with the infectious clones of CHIKV (isolate LR2006OPY1) [50], and ONNV (Chad strain) [39]. The CHIKV nsP genes were substituted for the corresponding ONNV genes using a restriction enzyme-based cloning method, and *vice versa*. The sequences of all plasmids were verified by Sanger sequencing, and virus stocks were obtained by transfection of *in vitro* transcribed RNA in Vero cells as described. ONNV UgMP30, IgJ10964, DakAr234, IbH12628, and SG650 (received from Center for Disease Control and Prevention) stocks were grown on Vero cells. ONNV nsP1 I386T, ONNV nsP1 M314 V, ONNV nsP3 G117R, and ONNV nsP3 *563R mutants were established from the ONNV RRC013 SG mCherry plasmid using Q5 Site-Directed Mutagenesis (New England Biolabs). The polymerase-negative ONNV nsP4 GAA (replication-deficient infectious clone) was previously described [51]. ONNV infectious RNA was developed by *in vitro* transcription (mMESSAGE mMACHINE SP6 Transcription Kit, ThermoFisher) of linearized plasmid (MssI restriction enzyme, ThermoFisher), and transfected (Lipofectamine3000, Invitrogen) into Vero cells. Virus titres were determined by end point dilution assay using a TCID_50_ assay on Vero cells.

### Infection and transfection of alphaviruses

For growth kinetic experiments, cells were infected at the indicated multiplicity of infection (MOI). Two hours post infection, cell monolayers were washed with phosphate-buffered saline (PBS), and medium was replaced with fresh culture medium. Medium samples were collected at several time points post infection to determine viral progeny by end point dilution assay on Vero cells.

Cells were transfected with *in vitro* transcribed infectious RNA using Lipofectamine 3000 (Invitrogen) in OptiMEM (Invitrogen). Two hours post transfection, medium was replaced with fresh culture medium. The cells were prepared for fluorescence-based visualization 24 h post transfection or flow cytometry at the indicated time points.

### Fluorescence-based visualization and flow cytometry

Cells were fixed in 4% paraformaldehyde (PFA) in PBS (Unimedic Pharma) for 30 min, permeabilized in 0.1% sodium dodecyl sulphate in PBS for 10 min, and incubated with blocking buffer (5% FBS in PBS) for 30 min. In case of antibody staining, cells were treated with 1:1000 primary anti-dsRNA mouse antibody J2 (SCICONS) or 1:2000 primary anti-CHIKV nsP1 rabbit antibody (received from Prof. Andres Merits, Institute of Technology, University of Tartu, Estonia) in blocking buffer for 1 h at room temperature, followed by respectively 1:2000 secondary anti-mouse Alexa 488 (Invitrogen) or 1:2000 secondary anti-rabbit Alexa Fluor 488 (Invitrogen) in blocking buffer for 1 h at room temperature. Cell monolayers were stained with 1:10000 Hoechst 33342 (Thermo Fisher) in PBS for 2 min. Between all steps, cells were washed and eventually incubated in PBS for visualization using the Cytation 5 cell imaging reader (BioTek).

Cells were detached in PBS, centrifuged and fixed in 4% PFA for 30 min. After two washing steps with PBS, analysis was performed with a ZE5 Cell Analyzer flow cytometer (Bio-Rad). Data was analysed using FlowJo.

### Infection of Lunet-HEK293 heterokaryons

HEK293 cells expressing the Doxycycline hyclate (DOX)-inducible Tet3G promoter were chemically fused with Lunet cells expressing the Tet3G-controlled ZsGreen1 reporter as described previously (54). 24 h post co-seeding of 1 × 10^5^ Lunet cells and 5 × 10^4^ HEK293 cells in a poly-D-lysine coated 24-well plate, cells were either treated with 125 µl pre-warmed 40% polyethylene glycol 1500 (PEG1500, Roche) fusion agent, or PBS as negative control. After 5 min incubation at 37°C, the PEG1500 was removed by extensive washing with PBS and fresh culture media with TET system-approved FBS (Gibco) and 0.5 µg/ml Doxycycline hyclate (DOX, D9891, Sigma-Aldrich). Cells were recovered for 24 h at 37°C before being infected with ONNV or CHIKV at a MOI of 0.1 or 1. 24 h post infection, cells were prepared for fluorescence-based imaging.

To allow high-throughput automated quantification of fluorescent cells, fluorescence images were computationally analysed by using the open-source image analysis tool CellProfiler 4.2.8. A customized pipeline was developed in which cell objects in the GFP and rhodamine channels were classified and overlay of the cell objects was quantified. This pipeline will be shared upon request.

### Mass spectrometry

Proteome analyses were performed by liquid chromatography-mass spectrometry (LC-MS) as described previously [52]. Of each cell line, 4–5 replicates from a 6-well format were provided for sample processing and analysis. Cells were detached with trypsin and washed with PBS. Thereafter, cells were lysed using RIPA buffer followed by cell debris removal by centrifugation at 12,000 × g and 4 °C for 10 min. 20 µg of proteins, as determined by BCA assays, were precipitated by ice-cold acetone (final concentration of 80%) followed by centrifugation at 11,000 × g and 4 °C for 15 min. In-solution digest was performed by Trypsin Gold (Promega) according to manufacturer’s instructions. Peptides were purified by self-made StageTips [53], dried in a SpeedVac vacuum concentrator, and resuspended in solvent A (0.1% formic acid, 5% acetonitrile in water (MS grade)).

LC-MS was performed on a Vanquish Neo nanoflow UHPLC System coupled to an Orbitrap Eclipse mass spectrometer (Thermo Scientific). 500 ng per sample were separated on a reversed-phase nanoViper PepMap column (150 mm length, 75 μm inner diameter, and 2 μm C18 particle size (Thermo Scientific)) at a flow rate of 250 nL/min at 40 °C with a gradient of solvent B (80% ACN, 0.1% FA) increasing from 6% to 25% within 60 min, further increasing to 90% within 10 min, and held at 90% for an additional 8 min. Peptides were ionized at 1900 V with a Nano Spray Flex Ion Source equipped with stainless steel emitters (40 mm, OD 1/32). Precursor scans were performed in the Orbitrap at a resolution of 120,000, followed by HCD fragmentation (collision energy set to 30%) of the 20 most intense precursors in the linear ion trap (dynamic exclusion set to 45 s). Fragment ions were scanned in the ion trap (normal mass range, maximum injection time set to auto, AGC target set to standard, and data type set to centroid).

Identification and quantification of proteins was performed with MaxQuant software (V. 2.4.2.0) [54] with preconfigured settings and additionally with label-free quantification via LFQ and IBAQ. MS spectra were searched against UniProt database of human proteins (ID UP000005640, reviewed, database downloaded on 04/03/2024). The resulting protein list was analysed with Perseus software (2.0.11, Max Planck Institute of Biochemistry) [55]. Proteins were removed if identified only by site modifications, identified as potential contaminants, or identified in the decoy database. Comparison of Lunet and HEK293 cells was further restricted to proteins detected in at least 3 out of 4 replicates in at least one cell line. Remaining proteins were grouped based on detection only in HEK293 cells, only in Lunet cells, or detection in both cell lines. In addition, the protein intensity of the HEK293-specific proteins was plotted against sequence coverage. For expression profiles of Huh7.5 cells, no further filters were applied.

### Immunoblots

Cell pellets were lysed in 1% Triton lysis buffer containing cOmplete Protease Inhibitor cocktail (Roche) (1 × 10^6^ cells/100 µl) for 30 min at 4°C. Samples were prepared in NuPage LDS Sample Buffer (ThermoFisher Scientific) with reducing agent (reduced conditions, ThermoFisher Scientific) or without reducing agent (non-reduced conditions), and heated to 95°C for 5 min. Proteins were separated on Bolt 4-12% Bis-Tris gels (ThermoFisher Scientific), and transferred onto PVDF membrane (Bio-Rad) by wet blotting. Membranes were blocked in PBS-Tween with 1% dry milk, and incubated in 1:100 primary α-CD81 (Invitrogen, non-reduced), 1:1000 primary α-FHL1 (R&D Systems, reduced) and 1:200 primary α-HSP70 (Santa Cruz, reduced and non-reduced) antibody overnight at 4°C, followed by incubation in 1:4000 secondary α-mouse HRP (ThermoFisher Scientific) antibody for 1 h at room temperature. Membranes were developed with HRP Substrate (Merck) for 2 min and signals were acquired through Amersham Imager 680.

### Viral passage assay

Mono-cultures of Vero, HEK293, Lunet, C6/36 and MSQ cells, and co-cultures of Vero and Lunet, and HEK293 and Lunet cells in 6-well cell culture plates were infected with ONNV RRC013 (MOI 0.1). Three to four days post infection, 500 µl supernatant was serially passaged to fresh mono- or co-cultures in 6-well cell culture plates, which was repeated for 5 passages. Supernatants of the individual passages were transferred to Lunet cells and screened for virus-induced cytopathic effect and viral mCherry expression. Samples that showed a positive result in passage five were harvested, titrated and used to infect Lunet cells at MOI 0.1 for viral growth kinetics analysis and sequencing.

### Illumina and Sanger sequencing

RNA was extracted from infected Lunet cells using the RNeasy Mini kit (Qiagen) and either prepared for Illumina or Sanger sequencing.

For Illumina sequencing, strand-specific cDNA libraries were generated and 2 × 150 bp Illumina sequenced (INVIEW Virus, Eurofins Genomics). The resulting reads were trimmed, quality-filtered, and mapped to the reference genome using Geneious Prime bioinformatic software. Multiple alignment comparisons of the consensus genomes were performed separately for the non-structural and structural genes' open reading frames using the default Geneious multiple alignment settings for translational alignment. To detect missense mutations in the ONNV genomes that were obtained after viral passaging, the “find variations/SNPs” function was used with a minimum variant frequency of 25%. To verify if the identified mutations were present in the ONNV WT stock, a minimum variant frequency of 1% was used.

For Sanger sequencing, extracted RNA was reverse transcribed and amplified in a reverse transcriptase-polymerase chain reaction (RT-PCR) by using the SuperScript III one-step RT-PCR system with platinum Taq DNA polymerase (Invitrogen) with ONNV-specific primers. The resulting RT-PCR products were visualized on a 1% agarose gel stained with GelRed, purified from gel (NucleoSpin Gel and PCR Clean-up, Macherey-Nagel), and Sanger sequenced (LightRun Tube, Eurofins Genomics).

### Strand-specific quantitative Real-Time PCR

RNA was extracted from infected Lunet cells using the NucleoSpin RNA mini kit for RNA purification (Macherey-Nagel). Random cDNAs and ONNV strand-specific cDNAs were transcribed using the High-capacity cDNA Reverse Transcription Kit (ThermoFisher) according to manufacturer’s protocol. The negative strand-specific cDNAs were generated using 5′-tagged forward primer (5′-GGCAGTATCGTGAATTCGATGCACGCGAGAAAACTTGCGTCA, Eurofins), and positive strand-specific cDNAs were generated using 5′-tagged reverse primer (5′-GGCAGTATCGTGAATTCGATGCTTTTTCCAGAGATGTTTTTATCTGT, Eurofins), based on [56]. Unincorporated primers were digested with Exonuclease I (ThermoFisher) by incubation at 37°C for 15 min followed by 85°C for 15 min. The GAPDH qPCR was performed with a set of GAPDH primers (5′-AGAAGGCTGGGGCTCATTTG and 5′-AGGGGCCATCCACAGTCTTC, Eurofins) and TaqMan probe with fam-BHQ1 (5′-AGCCAAAAGGGTCATCATCTCTGCCCC, Eurofins). The strand-specific qPCR was performed with a combination of strand-specific nsP1 primers with AT-rich 12-nucleotide flap sequence (positive strand qPCR 5′-AATAAATCATAATTTTTCCAGAGATGTTTTTATCTGT, and 5′-AATAAATCATAAGGCAGTATCGTGAATTCG ATGC, negative-strand qPCR 5′-AATAAATCATAAACGCGAGAAAACTTGCGTCA and 5′-AATAAATCATAAGGCAGTATCGTGAATTCGATGC, Eurofins) and TaqMan probe with fam-BHQ1 (5′-CCGCTGGAAAGGT, Eurofins) based on [56]. qPCR mixes (qPCRBIO Probe Mix Separate-ROX, PCRBiosystems) were prepared according to manufacturer’s protocol with 5 µl cDNA, and run on the QuantStudio 5 System (ThermoFisher) in duplicate with cycling conditions 95°C 2 min followed by (95°C 5 sec, 58-63°C 30 sec)×40.

### Statistical analysis

Graphical representation and statistical analysis were performed using GraphPad Prism 9. Results are presented as mean ± standard deviation for at least two independent experiments. Statistical differences were tested using two-way ANOVA with Šidák multiple comparison test, or one-way ANOVA with Dunnett’s multiple comparison test. The total number of replicates, statistical test, and significance levels are presented for each experiment.

## Results

### Lunet cells do not support ONNV RNA replication

**Figure 1.**
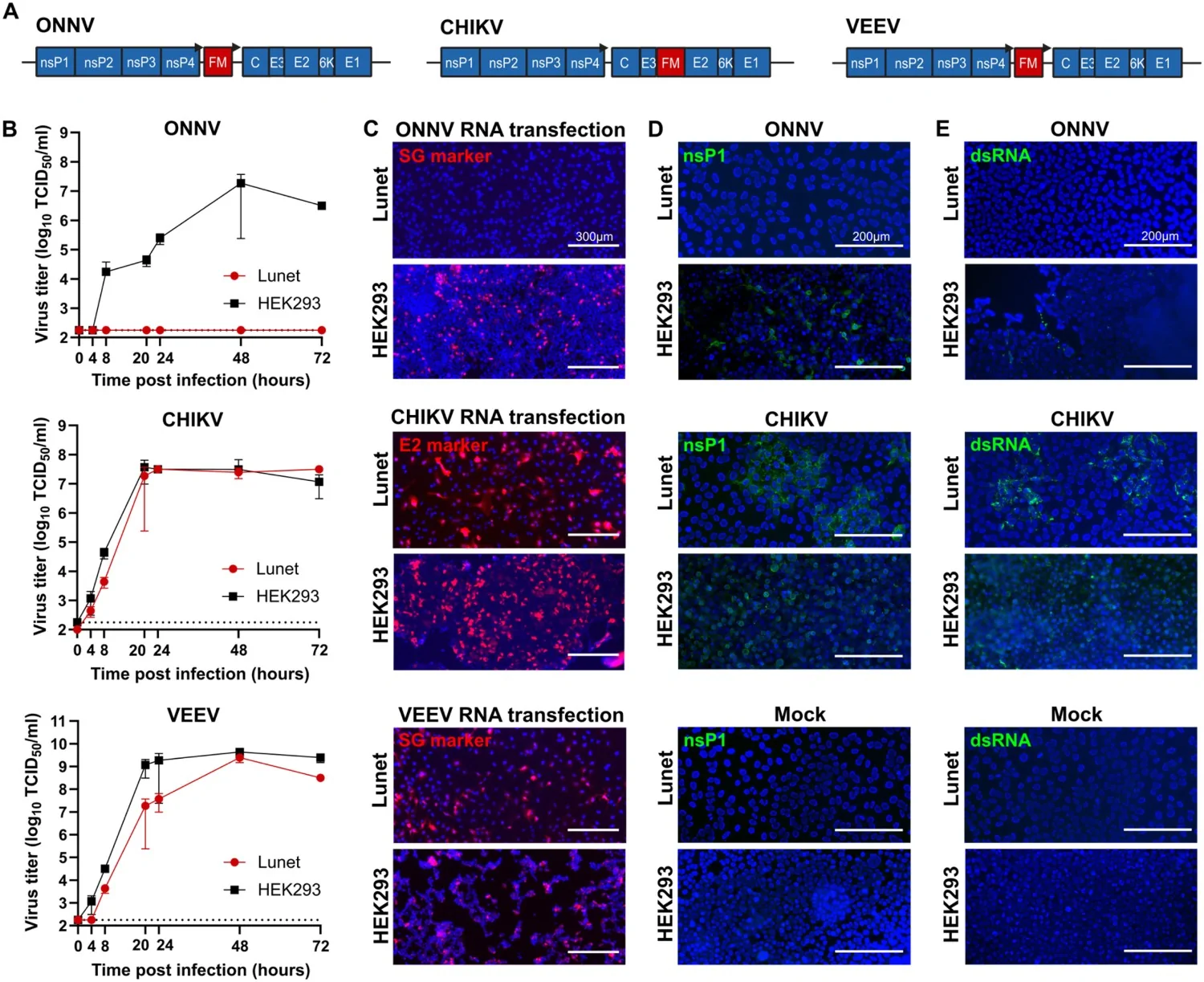
Lunet cells are non-permissive for ONNV infection. (A) Schematic of ONNV (RRC013 SG mCherry), CHIKV (37997 E2 mCherry), and VEEV (TC-83 SG GFP) infectious clones with fluorescent marker (FM). (B) Growth kinetics of ONNV, CHIKV, and VEEV in Lunet and HEK293 cells (MOI 0.1, n = 2, detection limit 1.78 × 10^2^ TCID_50_/ml indicated as dotted line). (C) Fluorescence microscopy of transfected ONNV, CHIKV, and VEEV infectious clones in Lunet and HEK293 cells at 24 h post transfection. (D,E) Fluorescence microscopy of ONNV, CHIKV, and mock-infected Lunet and HEK293 cells (MOI 0.1) at 24 h post infection. Cell nuclei stained with Hoechst, and virus infection visualized by (C) virus-encoded fluorescent marker (GFP recoloured), (D) anti-nsP1, and (E) anti-dsRNA immunofluorescent staining.

To better understand ONNV tropism determinants, we compared ONNV permissivity of different human cell lines. While ONNV (RRC013 SG mCherry) efficiently infected human embryonic kidney 293 (HEK293) cells, it was unable to form viral progeny in human hepatoma Huh-7-Lunet (Lunet) cells, which are clonal derivatives of Huh-7 cells selected for high hepatitis C virus replication and low CD81 expression (Figure 1(A,B)) [41]. In contrast, other alphaviruses, i.e. CHIKV (37997 E2 mCherry) and Venezuelan equine encephalitis virus (VEEV TC-83 SG eGFP), produced similar levels of viral progeny in Lunet and in HEK293 cells (Figure 1(A,B)). Next, we sought to determine at which ONNV life cycle step Lunet cells were refractory. Transfection of ONNV infectious RNA did not rescue infection in Lunet cells, whereas clear expression of the virus-encoded fluorescent marker was observed in ONNV transfected HEK293 cells. Moreover, Lunet cells supported replication of transfected CHIKV and VEEV genomic RNA (Figure 1(C)). This showed that ONNV infection was impaired at a post entry step, presumably at the RNA replication step of the viral life cycle (e.g. in the initial translation of the viral RNA, the formation of the replication complex, or RNA synthesis). To further investigate this hypothesis, we evaluated the presence of nsP1 and double stranded RNA (dsRNA), as indicators of nsP translation and active viral replication, respectively. In HEK293 cells, we observed ONNV RNA replication as early as 8 h post infection (hpi) by nsP1 and dsRNA detection using immunofluorescence staining (Supplementary Figure 1). In Lunet cells, however, we did not detect ONNV nsP1 and dsRNA, whereas we found CHIKV nsP1 and dsRNA at 24 hpi (Figure 1(D and E)). Taken together, ONNV RNA replication was compromised in Lunet cells, preventing infection.

### Absence of FHL1 limits ONNV infection in Lunet cells

**Figure 2.**
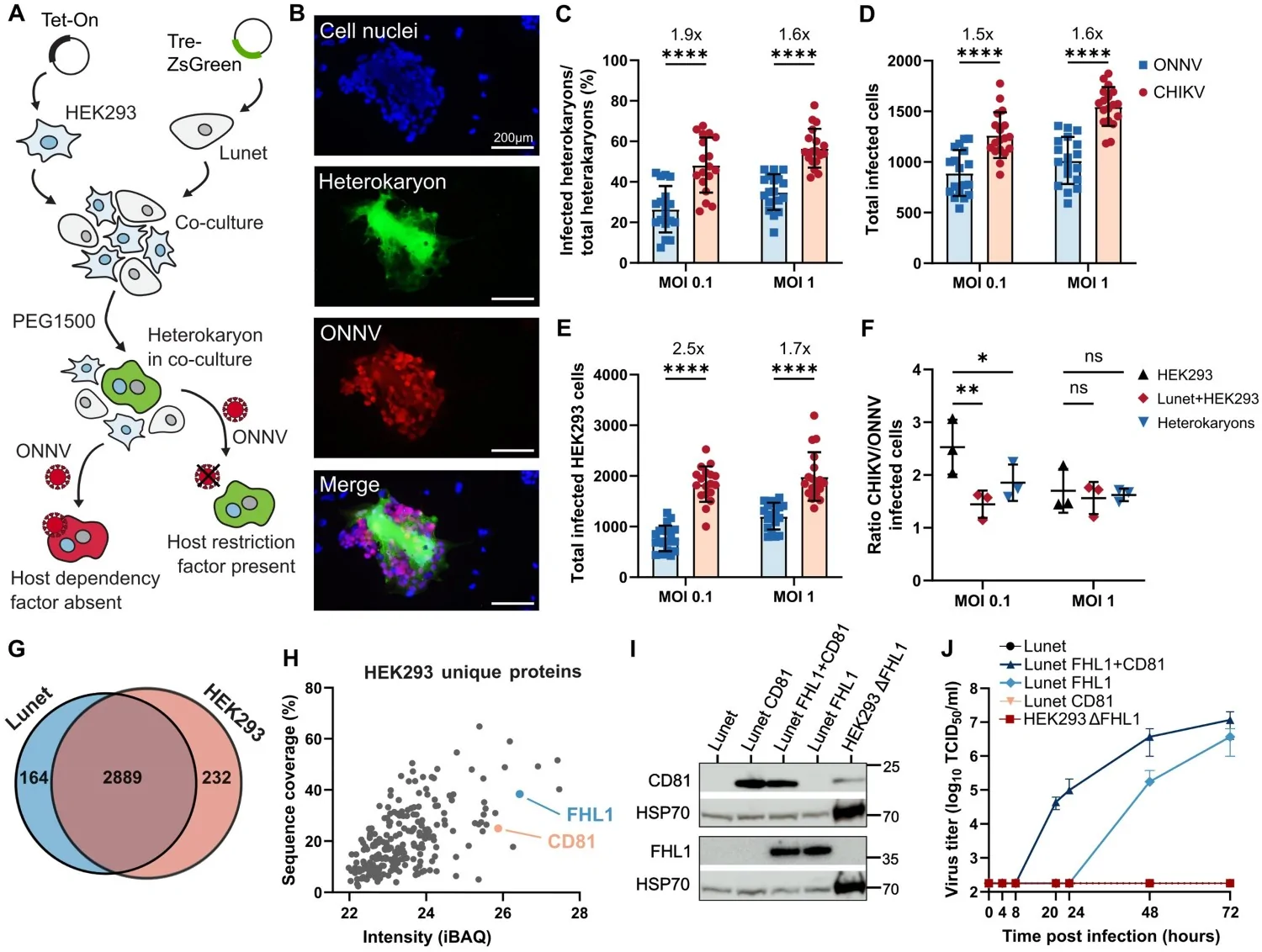
The absence of host dependency factors hinders ONNV infection in Lunet cells. (A) Schematic overview of the PEG1500-mediated cell fusion of HEK293-Tet-On and Lunet-Tre-ZsGreen co-cultures, and subsequent ONNV infection. (B) Fluorescence microscopy of ONNV-infected Lunet and HEK293 co-cultures and heterokaryons (MOI 0.1) at 24 h post infection. Cell nuclei stained with Hoechst, heterokaryon visualized by ZsGreen, and virus infection by mCherry. (C) The percentage of infected heterokaryons/total heterokaryons and (D) total number of infected cells, upon ONNV and CHIKV infection of Lunet-HEK293 heterokaryons in co-cultures (MOI 0.1 or 1, 24 h post infection). (E) The total number of ONNV and CHIKV infected cells in HEK293 monocultures (MOI 0.1 or 1, 24 h post infection). Bars represent the mean and standard deviation of three independent experiments with each six biological replicates. Ratios and significance between groups indicated above graph (two-way ANOVA, ****P < 0.0001). (F) Ratios of CHIKV:ONNV infected cells in the infected heterokaryons, Lunet and HEK293 co-cultures, and HEK293 mono-cultures. Bars represent the mean and standard deviation of three independent experiments with each six biological replicates. Significance between groups indicated above graph (two-way ANOVA, *P < 0.05, **P < 0.01). (G) Number of proteins identified in Lunet, HEK293, and both cell lines based on detection derived from shot gun proteomics (n = 4). (H) Average sequence coverage and intensity of the 232 HEK293-specific proteins as determined by shot gun proteomics. Median of biological quadruplicates shown. (I) Verification of stable expression of FHL1 and CD81 in Lunet cells and knock out of FHL1 in HEK293 ΔFHL1 cells by western blot of cell lysate using CD81, FHL1, and HSP70 (loading control) antibodies. (J) Growth kinetics of ONNV in Lunet, Lunet cells expressing human CD81, FHL1, or both CD81 and FHL1, and HEK293 ΔFHL1 cells (MOI 0.1, n = 2, detection limit 1.78 × 10^2^ TCID_50_/ml indicated as dotted line).

We hypothesized that the impaired ONNV RNA replication in Lunet cells is presumably caused by the absence of a host dependency factor or the presence of a host restriction factor. To investigate this, a Lunet-HEK293 heterokaryon assay was developed. Briefly, ONNV-susceptible HEK293 cells, expressing a Tet promoter (HEK293-Tet-On), were fused with ONNV-refractory Lunet cells, expressing a Tet-induced ZsGreen reporter (Lunet-Tre-ZsGreen) (Figure 2(A), Supplementary Figure 2(A)). Hence, fused cells express ZsGreen and are distinguishable from unfused cells. Fused cells were next infected with ONNV encoding an mCherry marker. Dual expression of mCherry and ZsGreen indicates that heterokaryons are permissive, likely because HEK293 complements the heterokaryon with a host dependency factor missing in Lunet cells. Alternatively, absence of dual expression indicates that heterokaryons are refractory, because a host restriction factor in Lunet cells inhibits heterokaryon infection (Figure 2(A)). In a subset of heterokaryons, dual expression was observed (Figure 2(B)), suggesting Lunet cells lack a host dependency factor. However, most heterokaryons expressed only ZsGreen, potentially due to the low multiplicity of infection (0.1 TCID_50_/cell) or unequal contribution of Lunet and HEK293 cellular material within heterokaryons.

To explore this further, we compared ONNV and CHIKV infectivity in Lunet-HEK293 heterokaryons. Although the total number of heterokaryons was similar in the ONNV and CHIKV infected cultures (Supplementary Figure 2(B)), the percentage of infected heterokaryons was significantly lower upon ONNV infection compared to CHIKV infection at MOI 0.1 and 1, respectively (Figure 2(C)). The total number of infected cells (both heterokaryons and non-fused cells) was also significantly lower for ONNV as compared to CHIKV in the heterokaryon assay (Figure 2(D)), as well as in HEK293 monoculture infections (Figure 2(E)). As the CHIKV:ONNV infection ratio did not increase in the heterokaryons compared to the HEK293 monocultures (Figure 2(F)), we concluded that ONNV’s reduced infectivity in heterokaryons was due to its overall lower infectivity and not due to a host restriction factor. This supports the hypothesis that Lunet cells lack a host dependency factor and that this lack is compensated by host factors from HEK293 cells in heterokaryons, allowing infection.

To assess which proteins are differentially expressed in Lunet versus HEK293 cells, we performed high-resolution quantitative shotgun proteomics by liquid chromatography coupled to mass spectrometry (LC-MS). A total of 2889 proteins were shared between Lunet and HEK293 cells, 164 proteins were unique to Lunet cells, while 232 were unique to HEK293 cells (Figure 2(G)). Among the most abundant unique HEK293 proteins, we found CD81 and FHL1 (Figure 2(H)). Both CD81 and FHL1 are known host factors for CHIKV and ONNV [34,36]. The transmembrane protein CD81 shapes cellular membranes and thereby facilitates alphavirus replication at the plasma membrane [34], while FHL1 interacts with the nsP3 hypervariable domain during RNA replication [36]. Depletion of either of these host factors results in inhibition of CHIKV and ONNV infection [34,36]. We therefore hypothesized that the absence of one or both host factors impaired ONNV infection in Lunet cells. To investigate this, we generated stable Lunet cell lines expressing CD81, FHL1, or both CD81 and FHL1, and evaluated expression using immunoblotting (Figure 2(I), Supplementary Figure 3). Individual supplementation of human CD81 did not rescue ONNV replication in Lunet cells, in contrast to human FHL1 supplementation (Figure 2(J)). Expression of both host factors resulted in a more rapid infection of Lunet cells. ONNV infection of another hepatocyte cell line (Huh7.5) that naturally lacks FHL1 and expresses CD81 did not result in viral progeny (Supplementary Figure 4). To validate the requirement for FHL1 in ONNV replication, CRISPR-based FHL1 knockout HEK293 cells (HEK293 ΔFHL1, Figure 2(I)) were infected with ONNV. As expected, no viral replication was detected (Figure 2(J)). In sum, the lack of FHL1 prevented ONNV replication in Lunet cells, while CD81 was not essential in this assay but supplementation had a positive effect on ONNV infection.

### ONNV laboratory strains and chimaeras carrying CHIKV nsP1-3 replicate in Lunet cells

**Figure 3.**
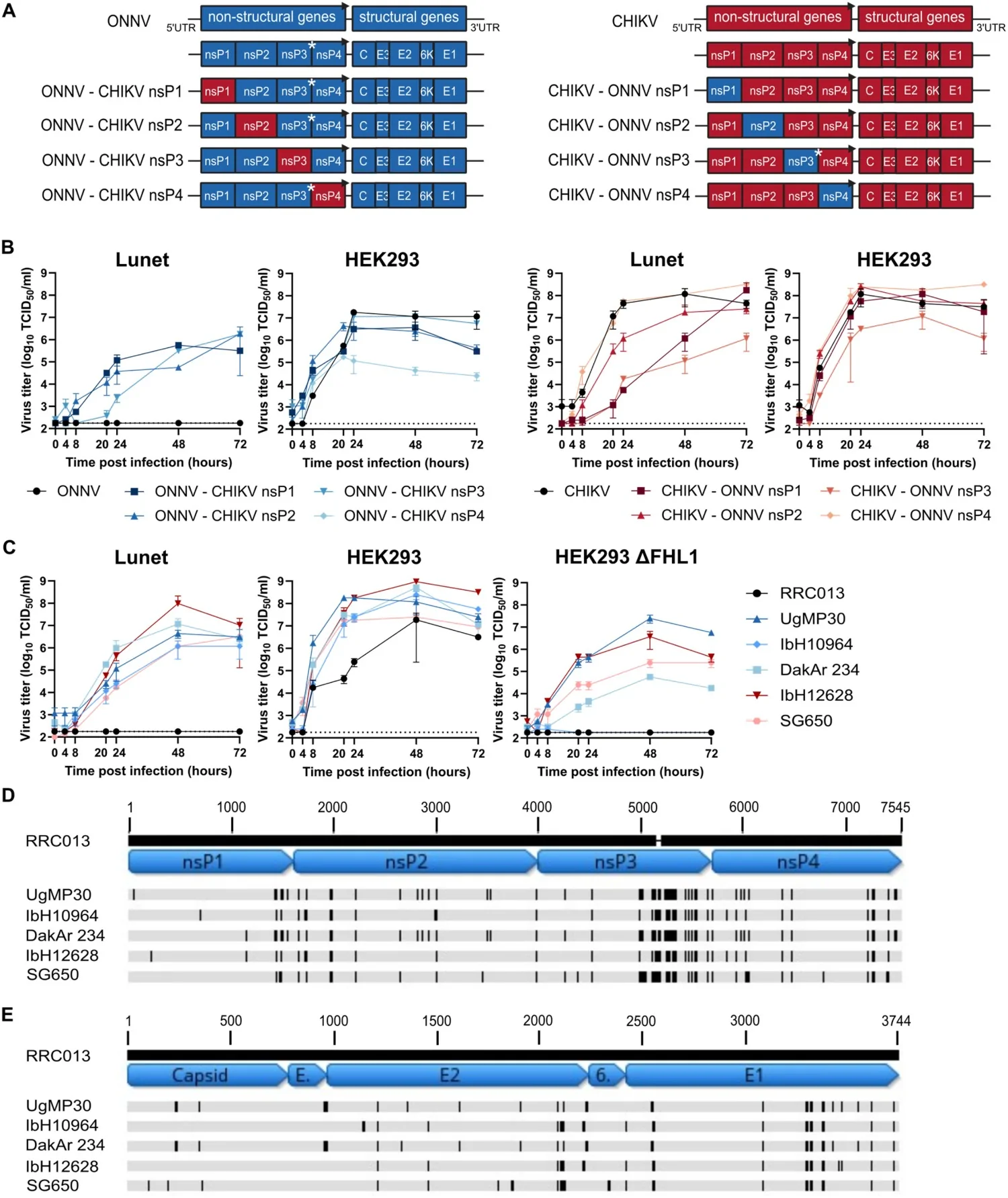
ONNV-CHIKV chimaeras and laboratory ONNV strains replicate in Lunet cells. (A) Schematic of ONNV, CHIKV, and ONNV-CHIKV chimaeras (asterix indicates opal stop codon, triangle indicates subgenomic promoter). (B) Growth kinetics of ONNV, CHIKV, and chimaeras in Lunet and HEK293 cells (MOI 0.1, n = 2, detection limit 1.78 × 10^2^ TCID_50_/ml indicated as dotted line). (C) Growth kinetics of multiple ONNV strains in Lunet, HEK293 and HEK293 ΔFHL1 cells (MOI 0.1, n = 2, detection limit 1.78 × 10^2^ TCID_50_/ml indicated as dotted line). (D,E) Multiple sequence alignment of Illumina-sequenced ONNV strains with difference to the ONNV RRC013 genome highlighted for (D) the non-structural genes and (E) structural genes.

ONNV and CHIKV are phylogenetically closely related, with ONNV RRC013 and CHIKV 37997 (West African lineage) sharing 88.1% sequence identity in their non-structural genes and 85.9% in their structural genes (based on pairwise distance computation in Mega12.0.14). Despite this similarity, differences in the non-structural genes, which govern RNA replication, may cause the replication defect of ONNV in Lunet cells. To evaluate this, we infected Lunet cells with ONNV and CHIKV chimaeras. In the ONNV-CHIKV chimaeras, individual non-structural genes were replaced with the corresponding CHIKV genes, and *vice versa* for the CHIKV-ONNV chimaeras (Figure 3(A)). Substitution of the CHIKV nsP1, nsP2 and nsP3 genes for the ONNV equivalents reduced virus replication in Lunet cells, while replacement of CHIKV nsP4 did not affect infection of Lunet cells (Figure 3(B)). In line with this, the *vice versa* substitution of the ONNV nsP1, nsP2 and nsP3 genes with the CHIKV equivalents rescued ONNV infection in Lunet cells, while exchange of the ONNV nsP4 for the CHIKV equivalent did not rescue replication in the Lunet cells and resulted in lower replication in the HEK293 cells (Figure 3(B)).

In addition, we compared infectivity of five ONNV strains in Lunet cells. Surprisingly, ONNV SG650, DakAr234, UgMP30, IbH10964 and IbH12628 all infected Lunet cells, and showed increased replication in HEK293 cells (Figure 3(C)). All strains, except IbH10964, were able to infect HEK293 ΔFHL1 cells, although replication was less efficient as in HEK293 cells (Figure 3(C)). These strains were collected as clinical isolates and have repeatedly been passed in mammalian cells to amplify virus stocks. In contrast, the ONNV RRC013 infectious clone has only been amplified once in cell culture and thereby represents a more original human clinical isolate. We hypothesized that passaging of ONNV strains in cell cultures allowed adaptation to mammalian cell lines like Lunet, while the ONNV RRC013 infectious clone is lacking the adaptive advantages.

To evaluate genomic differences, all ONNV strains were Illumina-sequenced and compared with the ONNV RRC013 genome (Figure 3(D and E)). Although multiple sequence variations were identified between the five ONNV strains and ONNV RRC013, a total of 36 amino acid residues in the RRC013 genome differed consistently from all other strains with 28 differences in the nsPs and 8 in the structural proteins (Supplementary Table 1). To narrow down potential differences associated to FHL1 dependency, the genomes of IbH10964 and IbH12628 were compared. In contrast to IbH12628, IbH10964 showed unable to replicate in HEK293 ΔFHL1 (Figure 3(C)), despite the two genomes differing by only six amino acids: four within the nsPs and two within the structural proteins (Supplementary Table 2). None of the amino acid differences between IbH10964 and IbH12628 showed the same residue in RRC013 and IbH10964 while differing from both IbH12628, SG650, DakAr234, and UgMP30. Based on these genomic comparisons, we could not determine which, if any, of the observed amino acid differences was responsible for adaptation to Lunet cells.

### Serial passaging of ONNV in mammalian and *Aedes albopictus* cells results in Lunet adaptation

**Figure 4.**
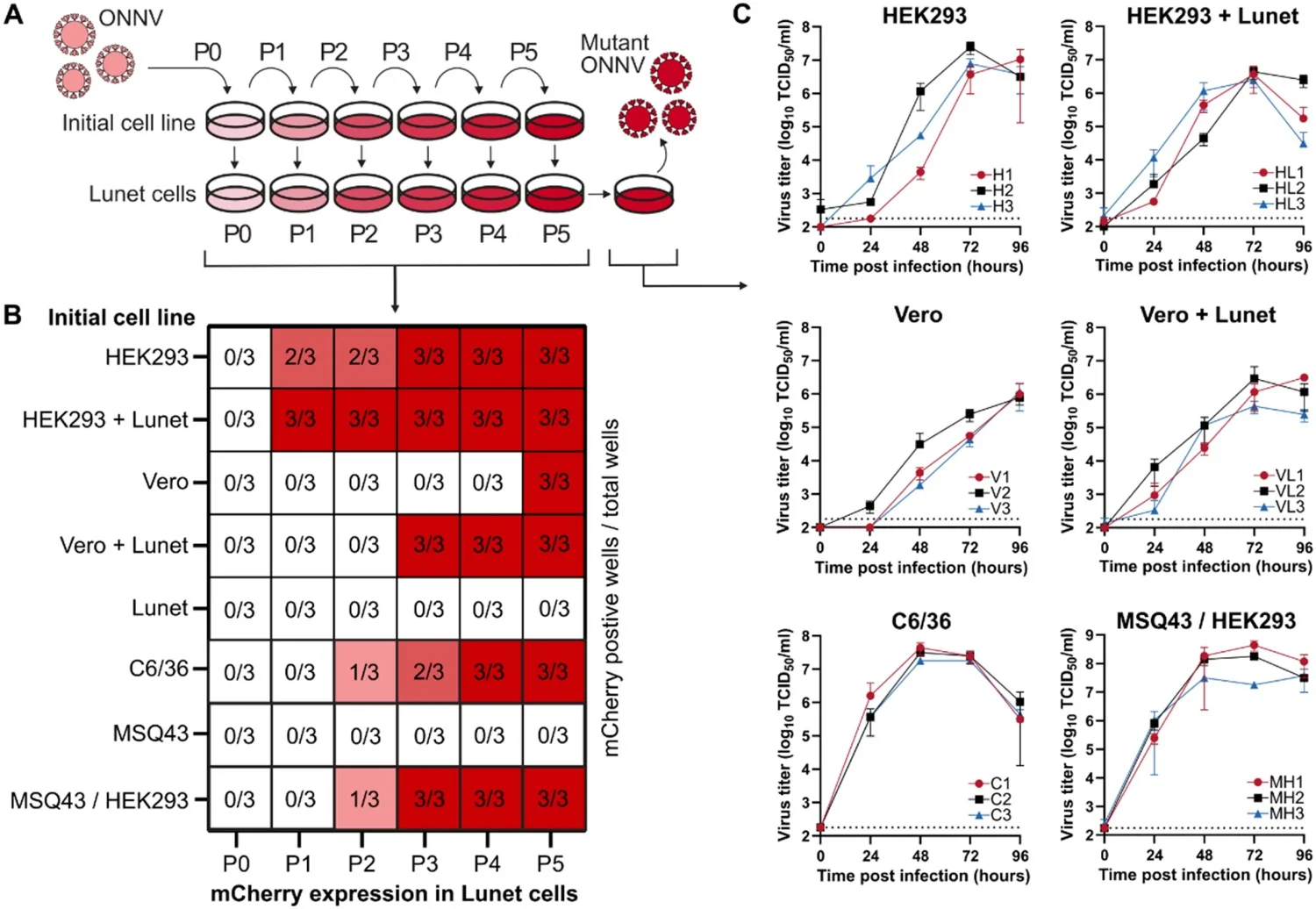
Serial passaging of ONNV in mammalian and *Aedes* cells results in Lunet cell adaptation. (A) Schematic representation of the serial passaging assay with passaging of ONNV on cells and verification of adaptive mutants by virus-encoded mCherry expression in Lunet cells. After five passages, mutants were isolated from Lunet cells. (B) Detection of ONNV mutants that express mCherry in Lunet cells after serial passaging in monocultures of HEK293, Vero, Lunet, C6/36 or MSQ43 cells, co-culture of HEK293 + Lunet cells or Vero + Lunet cells, or alternating passaging between MSQ43 and HEK293 cells. Figure represents a triplicate experiment with an initial MOI of 0.1. (C) Growth kinetics of isolated ONNV mutants in Lunet cells with indication of the passaging cell line (MOI 0.1, n = 2, detection limit 1.78 × 10^2^ TCID_50_/ml indicated as dotted line).

To examine the potential of ONNV adaptation, we serially passaged the virus and examined adaptation by expression of the ONNV-encoded mCherry marker (Figure 4(A)). After one passage in HEK293 mono-cultures and HEK293 + Lunet co-cultures, ONNV mutants were detected that expressed mCherry in Lunet cells (Figure 4(B)). These mutants persisted following serial passaging, were isolated after five passages, and demonstrated formation of progeny virus upon infection of Lunet cells at MOI 0.1 (Figure 4(C)). Serial passaging of ONNV in Vero mono-cultures and Vero + Lunet co-cultures resulted in Lunet-adapted mutants after five and three passages, respectively (Figure 4(B and C)). Mutants did not develop as rapidly as in HEK293 cells, presumably due to the absence of interferon signalling in Vero cells, which reduced selection pressure for cell culture adaptation [57]. In co-cultures, Lunet-adapted ONNV mutants had an advantage over ONNV wild-type (WT), resulting in earlier detection of ONNV mutants. Conversely, serial passaging in Lunet mono-cultures did not result in adapted mutants, as initial RNA replication was unsuccessful (Figure 4(B)).

To assess whether adaptive mutations were host species dependent, we passaged ONNV in mosquito cell cultures. In C6/36 cells (*Aedes albopictus*), we detected Lunet-adapted mutants after two to four passages (Figure 4(B and C)), while we did not find such mutants in MSQ43 cells (*Anopheles stephensi*) upon passaging. In the latter, which is a cell line derived from the same mosquito genus as the main transmission vector, ONNV vanished after two passages as proliferation was inefficient in MSQ43 cells (Figure 4(B) and Supplementary Figure 5(A)). Alternating passaging between MSQ43 and HEK293 (initial infection in MSQ43) resulted in higher virus titres and ONNV-adapted mutants (Figure 4(B and C)). Summarizing, serial passaging of ONNV in mammalian and some mosquito cells resulted in mutants with altered tropism, allowing Lunet cell infection.

### Single mutations in ONNV nsP1 and nsP3 overcome FHL1 deficiency

**Figure 5.**
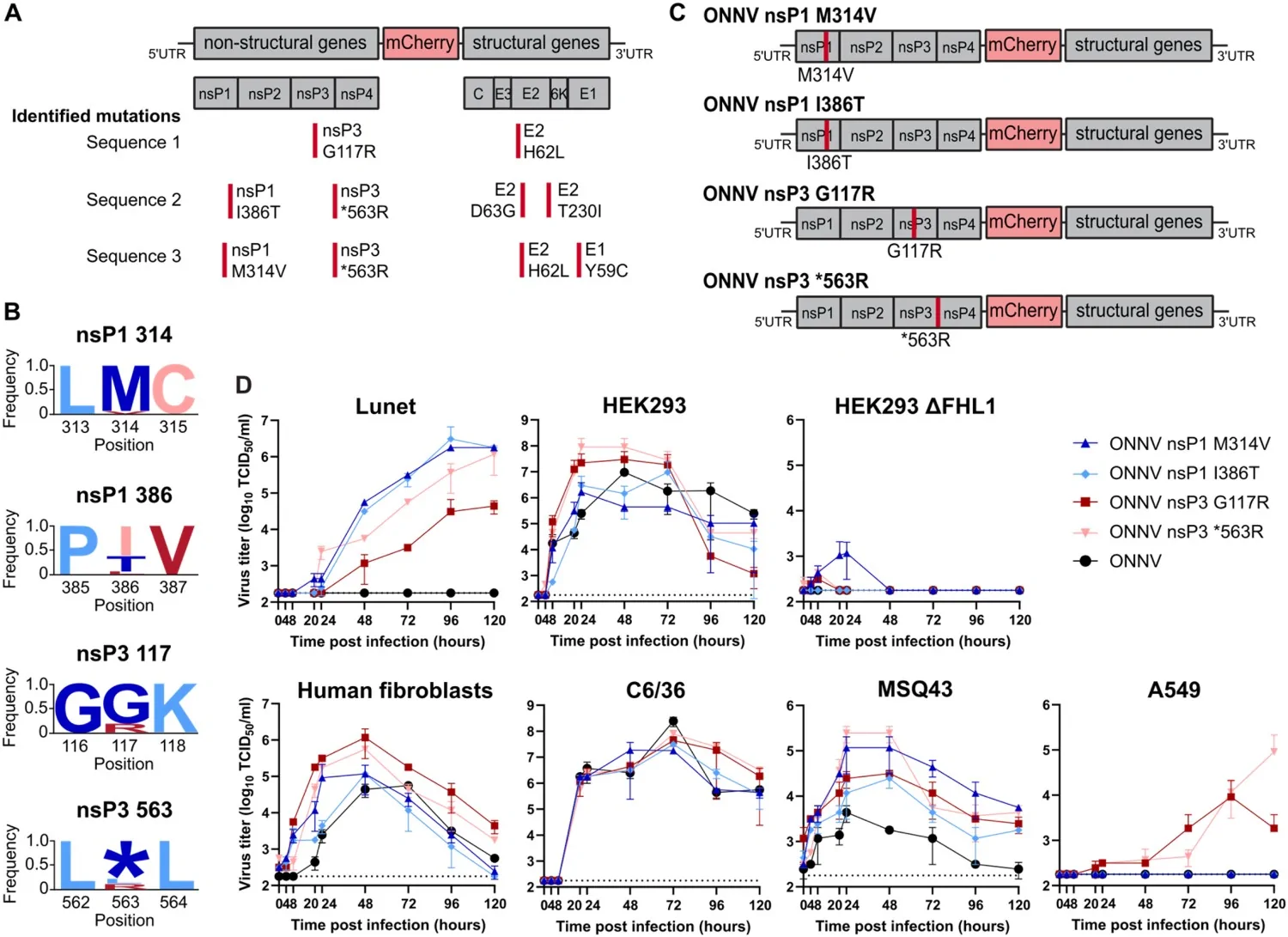
Single ONNV nsP1 and nsP3 mutations result in infection of Lunet and A549 cells. (A) Missense mutations identified by Illumina RNA sequencing of three Lunet-adapted ONNV mutants: sequence 1 was derived from ONNV isolate passaged in HEK293 monoculture, sequence 2 from HEK293 + Lunet co-culture, and sequence 3 from Vero monoculture. (B) Verification of nsP mutations in the 18 isolated ONNV mutants by Sanger sequencing (nsP1 314 (17xM, 1xV), nsP1 386 (11x I, 6xT, 1xL), nsP3 117 (15xG, 3xR), nsP3 563 (14x*, 2xR, 2xL), with asterisks referring to the opal stop codon). (C) Schematic representation of the site-directed mutagenesis generated ONNV mutants. (D) Growth kinetics of ONNV mutants in Lunet (human hepatoma), HEK293 (human embryonic kidney), HEK293 ΔFHL1, human fibroblasts, C6/36 (*Aedes albopictus*), MSQ43 (*Anopheles stephensi*), and A549 (human lung epithelial) cells (MOI 0.1, n = 2, detection limit 1.78 × 10^2^ TCID_50_/ml indicated as dotted line).

Serial passaging yielded 18 Lunet-adapted ONNV mutant strains, three of which were analysed by Illumina sequencing (Figure 5(A)). Missense mutations were detected in both non-structural and structural genes. We focused on the non-structural mutations, as their role in RNA replication is directly relevant to the impaired replication of ONNV in Lunet cells. The first Illumina-sequenced mutant displayed only one mutation in the non-structural genes, i.e. nsP3 G117R, located in the nsP3 macrodomain (Figure 5(A)). The exact same mutation was detected in two other mutants by Sanger sequencing (Figure 5(B)), and present in 2% of the Illumina-sequenced ONNV WT stock reads (Supplementary Figure 5(B)). The second and third Illumina-sequenced mutants presented mutations in nsP1 (nsP1 M314 V and nsP1 I386 T) and nsP3 (nsP3 *563R, with asterisks referring to the opal stop codon) (Figure 5(A)). The nsP1 mutations, both in the ring-aperture membrane-binding and oligomerization (RAMBO) domain, clustered in close proximity within the protein structure (M314 in β-sheet β13 and I386 in α-helix αj [21], Supplementary Figure 5(C)), whereas the nsP3 opal stop codon mutation was identical in both isolates. The nsP1 mutation at position 314 was not detected in any of the other 15 mutants, while mutations at nsP1 position 386 appeared six additional times (five times I386 T and once I386L), and at nsP3 position 563 twice more (as *563L) (Figure 5(B)). These mutations were not present in the ONNV WT stock (Supplementary Figure 5(B)). Of all identified mutations in the non-structural genes, the threonine at position 386 in nsP1 was identified in the IbH12628 and DakAr234 genomes, and the arginine at position 563 in nsP3 was present in all five previous described ONNV strains (Supplementary Table 1 and 2). Overall, this indicates that several different mutations in the ONNV nsP1 and nsP3 genes were developed during passaging of the ONNV WT and resulted in extended tropism.

ONNV infectious clones were generated with the previously Illumina-sequenced nsP mutations to access the influence of single mutations on Lunet cell infection (Figure 5(C)). All single mutations successfully facilitated ONNV infection of Lunet cells (Figure 5(D)). The ONNV nsP1 mutants exhibited superior replication in Lunet cells than the nsP3 mutants. At 120 h, the ONNV nsP3 *563R mutant reached virus titres equivalent to the nsP1 mutants. In contrast, the ONNV nsP3 G117R mutant showed lower replication in Lunet cells than the other mutants throughout the whole time course. In HEK293 cells, both ONNV nsP3 mutants replicated more efficiently than ONNV WT, while the ONNV nsP1 mutants showed similar growth kinetics as the WT virus. Although the single mutations restored replication capacity in FHL1-deficient Lunet cells, they failed to enable infection of FHL1-deficient HEK293 cells, likely reflecting cell-type-specific differences in expression of other host factors.

To evaluate if these mutations compromised infection in other cells, growth kinetics were also assessed in a variety of other mammalian and mosquito cells (Figure 5(D)). Similar replication patterns were observed in human fibroblast cells as in HEK293 cells with the ONNV nsP3 mutants replicating more efficiently than ONNV WT and the nsP1 mutants. In C6/36 cells, all ONNV mutants replicated comparably to ONNV WT. Conversely, in MSQ43 cells, all mutants outcompeted ONNV WT, with ONNV nsP3 *563R and ONNV nsP1 M314 V reaching highest virus titres. Virus titres plateaued for all tested mutants and ONNV WT between 24 and 48 hpi and declined up to 120 hpi. In sum, ONNV mutants showed enhanced replication in mammalian cells and in cells derived from its main transmission vector genus *Anopheles*.

To assess whether the identified nsP1 and nsP3 mutations conferred broader adaptation, another non-permissive cell line was infected with the generated ONNV mutants. Like Lunet cells, the human lung epithelial cell line A549 is non-permissive for ONNV WT infection (Figure 5(D)). Although the ONNV nsP1 mutants were unable to infect A549 cells, both nsP3 mutants established delayed infections (Figure 5(D)). Thus, ONNV nsP1 mutations M314 V and I386V were more Lunet cell specific, while the ONNV nsP3 G117R and *563R mutations enhanced viral replication in mammalian and in *Anopheles* cells.

### Adaptive ONNV nsP1 and nsP3 mutations enhance RNA replication

**Figure 6.**
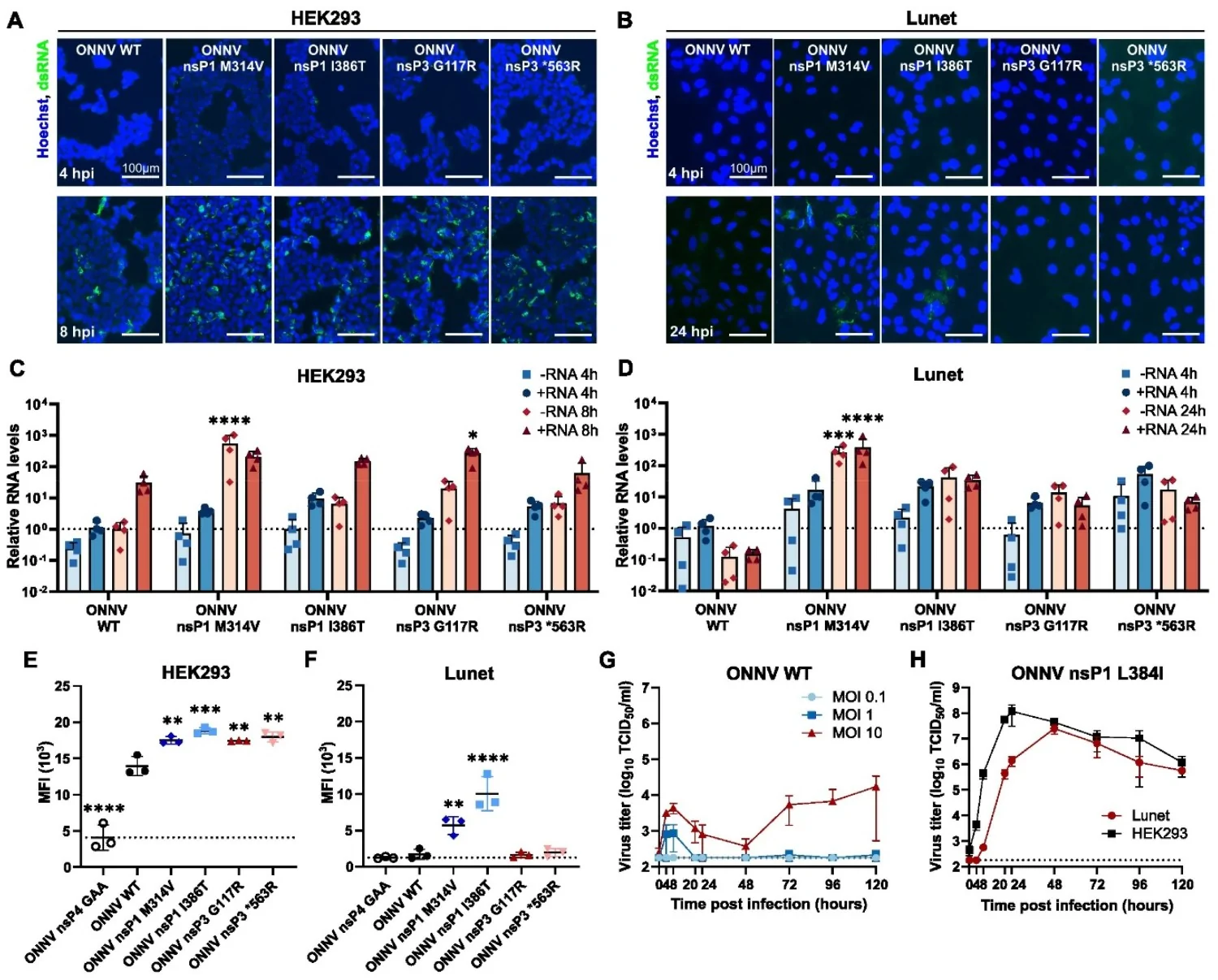
ONNV nsP mutations enhance RNA replication. (A,B) Fluorescence microscopy of ONNV WT and mutants in (A) HEK293 and (B) Lunet cells (MOI 0.1). Cell nuclei stained with Hoechst, and virus infection visualized by anti-dsRNA immunofluorescent staining. (C,D) Negative- and positive-strand RNA quantification in (C) HEK293 and (D) Lunet cells upon ONNV WT and mutant infections (MOI 0.1) based on strand specific qRT-PCR. RNA levels were normalized to GAPDH and presented relative to ONNV positive-strand RNA at 4 hpi. Bars represent the mean and standard deviation (n = 4, two-way ANOVA, ****P < 0.0001, ***P < 0.0005, *P < 0.05). (E,F) Flow cytometry quantification of subgenomic encoded mCherry expression following transfection of ONNV WT, ONNV mutants, and a replication deficient ONNV (ONNV nsP4 GAA, negative control) infectious clone. (E) Mean fluorescence intensity (MFI) in HEK293 at 12hpt and (F) Lunet cells at 24hpt. Bars represent the mean and standard deviation (n = 3). Asterisks indicate statistically significant differences compared with ONNV WT (one-way ANOVA, ****P < 0.0001, ***P < 0.0005, **P < 0.001, and *P < 0.05). (G) Growth kinetics of ONNV infection in Lunet cells at MOI 0.1, 1, and 10 (n = 4, detection limit 1.78 × 10^2^ TCID_50_/ml indicated as dotted line). (H) Growth kinetics of site-directed mutagenesis-derived ONNV nsP1 L384I mutant in Lunet and HEK293 cells (MOI 0.1, n = 2, detection limit 1.78 × 10^2^ TCID_50_/ml indicated as dotted line).

The elevated virus titres in mammalian and mosquito cells, combined with the capacity to overcome RNA replication restrictions in Lunet cells, suggested that the identified ONNV mutants acquired a more efficient RNA replication machinery. To evaluate potential differences in RNA replication, we quantified viral negative- and positive-strand RNA in HEK293 and Lunet cells following infections (Figure 6(A and B)), and visualized dsRNA complexes using immunofluorescent staining (Figure 6(C and D), Supplementary Figure 6 (A and B)). Viral RNA quantification and visualization were acquired early in infection, at 4 hpi, and during the release of the first viral progeny, i.e. at 8 and 24 hpi for HEK293 and Lunet cells, respectively.

In the HEK293 cells, viral RNA replication increased over time for ONNV WT and mutants, as shown by dsRNA staining and quantification of negative- and positive-strand RNA (Figure 6(A and C)). At 4 hpi, only a few dsRNA complexes were detected, whereas by 8 hpi dsRNA complexes were detected in numerous cells, corresponding to the increase in negative- and positive-strand RNA (Figure 6(A)). At this late time point in the infection cycle, the ONNV mutants showed higher levels of both RNA strands compared to ONNV WT, although significant increases were only observed for ONNV nsP1 M314V negative-strand RNA and ONNV nsP3 G117R positive-strand RNA (Figure 6(C)).

In the Lunet cells, negative- and positive-strand RNA levels increased during ONNV mutant infections, while ONNV WT RNA levels declined (Figure 6(B and D)). Most mutants showed higher negative- and positive-strand RNA levels than ONNV WT at 4 hpi, and by 24 hpi all mutants showed elevated levels compared to ONNV WT (Figure 6(D)). However, only ONNV nsP1 M314 V exhibited significantly higher RNA levels, consistent with the prominent detection of dsRNA (Figure 6(B)).

Notably, in Lunet cells the levels of negative- and positive-strand RNA were nearly equal, whereas in HEK293 cells, positive-strand RNA was more abundant (Figure 6(C and D), Supplementary Figure 6 (A,B)). Typically in alphavirus RNA replication, a single negative-strand RNA serves as template for multiple positive-strand RNAs, roughly 1:100 [16]. In Lunet cells, however, this ratio was altered, potentially reflecting the absence of CD81 and FHL1 (Supplementary Figure 6(A,B)).

To confirm that the enhanced RNA replication is independent of virus entry, *in vitro*-transcribed infectious RNA was transfected into HEK293 and Lunet cells. Replication was assessed by subgenomic-encoded mCherry gene expression using flow cytometry. In HEK293 cells, mCherry was detected as early as 12 h post transfection (hpt) (Supplementary Figure 6(C)). The mean fluorescence intensity was significantly higher in cells transfected with ONNV mutants compared to ONNV WT (Figure 6(E)), indicating enhanced replication independent of virus entry. In Lunet cells, mCherry was only detected for the ONNV nsP1 M314V and ONNV nsP1 I386T mutants as early as 24 hpt, in contrast to the other mutants and ONNV WT (Figure 6(F), Supplementary Figure 6(D)), indicating the advantage of the nsP1 mutations in Lunet cells (Figure 5(D)).

Given the enhanced RNA replication of ONNV mutants, we examined whether the RNA replication deficiency in Lunet cells could be bypassed by delivering more infectious RNA to the cells. Infections with ONNV WT at a MOI of 1 and 10 showed a short-lived increase in viral titres during the initial 8 hpi (Figure 6(G)). Subsequently, viral titres decreased, reaching undetectable levels for the MOI 1 infection by 20 hpi, and low levels for the MOI 10 infection by 48 hpi. Remarkably, three of the four MOI 10 replicates showed detectable viral growth after 48 h (Supplementary Figure 6(E)). From these replicates, viral RNA was isolated at 120 hpi and sequenced. All three isolates presented the same nsP1 mutation, L384I, which was only two amino acids apart from the previously identified mutation in the nsP1 RAMBO domain (nsP1 I386T). To confirm that nsP1 L384I mediates growth in Lunet cells, an ONNV nsP1 L384I mutant was generated, showing robust growth in both Lunet and HEK293 cells (Figure 6(H)). Taken together, higher input RNA concentrations allowed RNA replication in Lunet cells, yet this advantage wanes unless a mutant capable of spreading to uninfected Lunet cells emerged.

## Discussion

Here, we describe how FHL1 deficiency in Lunet cells can be overcome by single nucleotide mutations in the ONNV genome that enhance RNA replication. These mutants arose during serial passaging of ONNV RRC013 in mammalian and mosquito (*Aedes*) cells and appeared quicker in cells with a higher cellular barrier (HEK293, and co-cultures with Lunet cells) than in cells with a lower cellular barrier (Vero and C6/36 cells). Passaging in cells derived from the main mosquito vector genus (*Anopheles*) did not result in adaptation to Lunet cells. Moreover, high initial virus titres led to temporary infection that could transition into permanent replication once an adapted mutant arose. The ONNV nsP1 mutants were most abundant and demonstrated advantageous in overcoming the Lunet cell restriction, exhibiting superior replication over the other mutants. In contrast, the ONNV nsP3 mutants conferred a broad replication advantage across multiple mammalian and mosquito cells, even overcoming restriction in non-permissive A549 cells. Despite their ability to infect FHL1 deficient Lunet cells, theses mutants failed to efficiently replicate in FHL1 deficient HEK293 cells, indicating that the enhancement of RNA replication is dependent on the cellular context and does not uniformly result in overcoming the same host factor.

All identified nsP1 mutations were positioned in the RAMBO domain which composes, together with the capping domain, the nsP1 ring complex. The capping domain forms the crown of the complex, while the RAMBO domain anchors the complex in the plasma membrane and forms the pore, which presumably interacts with nsP4 [21]. While nsP1 M314V was located in β-sheet β13, involved in membrane binding, the nsP1 I386T and L384I mutations were located in α-helix αj, which forms together with two other helices the inner wall of the pore [21]. Other studies also linked mutations in these regions to the ability of alphaviruses to overcome replication barriers in cells. SINV nsP1 I351V, I388V, and N374H suppressed a negative-strand RNA synthesis defect in nsP4 [58], CHIKV nsP1 M314V allowed infection in non-permissive A549 cells [59], and CHIKV nsP1 G230R and V326M were able to overcome polyamine depletion by optimized membrane binding and increased synthesis of capped positive-strand RNA, respectively [60]. This is consistent with our observations that mutations in the RAMBO domain enhance RNA replication, potentially linked to improved membrane anchoring, or fine-tuned nsP4 interaction.

Studies on the functioning of alphavirus nsP3 in the RNA replication complex mainly focus on the nsP3 hypervariable domain. The latter shows variation among alphaviruses with regards to its binding of essential host proteins involved in RNA replication, like FHL1 [27,36,61]. In contrast to the hypervariable domain, the nsP3 macrodomain is conserved, but similarly essential in the RNA replication complex. First, the macrodomain initiates the replication complex by ADP-ribose binding; second it amplifies replication by hydrolysing ADP-ribose from monomeric ADP-ribose(MAR) proteins [62,63]. A study on the nsP1 Y114A macrodomain mutation in CHIKV demonstrated increased ADP-ribose binding activity for this mutant, resulting in more rapid nsP translation and dsRNA formation [62]. The same mutation in SINV demonstrated increased viral replication in mice [64]. We hypothesize that the identified ONNV nsP3 G117R mutation causes a similar enhancement in ADP-ribose binding activity, thereby overcoming the limitation in Lunet cells during early RNA replication, and additionally resulting in a significant increase in positive strand RNA synthesis in HEK293 cells.

The variation in stop and sense codons at the end of nsP3 has been described for various laboratory strains and natural isolates of alphaviruses [60,65]. The stop codon orchestrates the translational readthrough of the nsP1234 polyprotein to optimize the production of nsP4 at a rate of roughly 5–20% [32]. Replacement of the opal stop codon by a sense codon results in continuous production of nsP4 and a delay in polyprotein processing [16]. In detail, the overproduction of nsP1234 delays nsP1–nsP2 cleavage, thereby postponing the transition from negative- to positive-strand RNA synthesis. This results in negative-strand RNA overproduction, while genomic RNA is reduced as nsP2–nsP3 cleavage (responsible for the transition of genomic to subgenomic RNA synthesis) is not delayed [16]. A natural delay in replication reduces the disadvantage of the sense codon, for example during replication in mosquitoes at 28°C or by a delayed nsP1–nsP2 cleavage motif [16]. Accordingly, additional factors in viral replication might explain the contrasting findings that some sense codon variants reduced infection [15,60], while in other circumstances no difference or even improved infection was observed [15,66–70]. Here, the ONNV sense codon, with its presumably increased nsP4 production, had a general cell culture advantage and was especially beneficial in circumstances where RNA replication was restricted.

Our findings highlight that even subtle genomic changes in alphaviruses can profoundly influence viral replication and fitness. During alphavirus RNA replication, mutants naturally develop and persist even without selection pressure. In environments with limited cellular resources, such as in Lunet cells, variants with enhanced replication capacity become dominant. We observed that different mutations in alphavirus nsPs lead to a comparable enhancement in RNA replication, but likely through distinct mechanisms. The nsP1 mutants probably involved improved nsP1 ring structure interactions, which was especially beneficial in Lunet cell infection. Whereas more broad tropism advantages were observed with the nsP3 macrodomain mutation, which presumably involved ADP-ribose binding activity, and with the nsP3 stop to sense codon mutation, which changes the nsP processing and thereby RNA replication. NsP mutations not only facilitate alphavirus adaptation to new environments, as shown here and by others [59], but they can also result in increased epidemic transmission [71], or hamper the efficiency of antivirals treatments [60,72]. Interestingly, the here described mutations did not occur upon passaging in *Anopheles* cells and did not confer an advantage in *Aedes* cells. This may indicate that during the natural transmission cycle between mosquitoes and mammals, such mutations would not become fixed. We are currently investigating the molecular pathways behind the distinct adaptation mechanisms. Further insights into the interplay between alphaviruses replication kinetics, host cell innate immunity and host dependency factors will help refining the determinants of virus specificity and prediction of the evolutionary adaptation landscape shaping tropism and antiviral resistance.

## Supplementary Material

Supplementary file.docx
