# Supplementary material for: O’nyong-nyong virus adaptive mutations in non-structural protein 1 and 3 enhance RNA replication and overcome FHL1 requirement
Source: Emerg Microbes Infect. 2026 Aug 19;15(1):2721742. doi: 10.1080/22221751.2026.2721742 (PMC13528125; doi:10.1080/22221751.2026.2721742)
Supplement: Supplementary file 1 [file TEMI_A_2721742_SM2875.docx]

**Supplementary figures**

**Supplementary figure 1** Fluorescence microscopy of ONNV and CHIKV infected Lunet and HEK293 cells (MOI 0.1) at 4 and 8 hpi. Cell nuclei stained with Hoechst, and virus infection visualized by anti-nsP1, and anti-dsRNA immunofluorescent staining.

**Supplementary figure 2** (A) Fluorescence microscopy of ONNV infected Lunet and HEK293 co-cultures and heterokaryons (MOI 0.1) at 24 hours post infection. Cell nuclei stained with Hoechst, heterokaryon visualized by ZsGreen, and virus infection by mCherry. (B) Total number of heterokaryons, upon ONNV and CHIKV infection of Lunet-HEK293 heterokaryons in co-cultures (MOI 0.1 or 1, 24 hours post infection). Bars represent the mean and standard deviation of three independent experiments with each six biological replicates. Significance between groups indicated above graph (two-way ANOVA).

**Supplementary figure 3** (A) Western blot verification of CD81 and FHL1 expression in Lunet cells and loss of FHL1 in HEK293 ΔFHL1 cells using CD81, FHL1, and HSP70 (loading control) antibodies. (B) Quantification of CD81 and FHL1 in Lunet, Lunet CD81 (CD81), Lunet FHL1+CD81 (FHL1+CD81) and Lunet FHL1 (FHL1) cells normalized to HSP70 on western blot (n=2) using ImageJ software.

**Supplementary figure 4** ONNV restriction in Huh7.5 cells due to the absence of FHL1. (A) Growth kinetics of ONNV in HEK293, Huh7.5, and Lunet cells (MOI 0.1, n=2, detection limit 1.78x10^2^ TCID_50_/ml indicated as dotted line). (B) LC-MS based whole cell proteome analysis of Huh7.5 cells with intensities of CD81 and FHL1 of each replicate (iBAQ, log2; blue/red = low/high intensity). Grey boxes indicate replicates in which CD81 and FHL1 were not detectable. (C) Median intensities (iBAQ, log2, n=5) and sequence coverage (%) of all protein identified.

**Supplementary figure 5** (A) Schematic representation of detected mCherry expression and CPE in Vero cells after serial passaging in monocultures of MSQ43 cells. Figure represents triplicate experiment with an initial virus infection of MOI 0.1. (B) Missense mutations in the non-structural genes identified by Illumina RNA sequencing of three Lunet-adapted ONNV mutants upon viral passaging, and verification of the presence of these mutations in the ONNV WT stock prior to viral passaging. The percentage of reads that showed the detected mutation with a detection limit of 1% (BDL indicating below the detection limit) and the coverage at this nucleotide are presented. (C) Alphafold 3 modelling of non-structural protein 1 with indication of M314 (highlighted in green) in the β-sheet 13 and I386 (highlighted in pink) in α-helix J.

**Supplementary figure 6** (A,B) Ratios of positive- vs negative-strand RNA in (A) HEK293 and (B) Lunet cells upon ONNV WT and mutant infections (MOI 0.1) based on strand specific qRT-PCR. Bars represent the mean and standard deviation (n=4, two-way ANOVA, *P < 0.05). (C,D) Flow cytometry quantification of mCherry positive cells following transfection of ONNV WT and mutant infectious clones. (C) Percentage of mCherry positive singlets in HEK293 cells at 12 hpt. (D) Percentage of mCherry positive singlets in Lunet cells at 24hpt. Bars represent the mean and standard deviation (n=3). (E) Growth kinetics of ONNV infection in Lunet cells at MOI 10 as presented in Figure 6G shown separately for each sample (detection limit 1.78x10^2^ TCID_50_/ml indicated as dotted line).

| **Gene** | **Amino acid** | **RRC013** | **Other strains** |
| --- | --- | --- | --- |
| nsP1 | 483 | Y | H |
|  | 496 | N | D |
| nsP2 | 21 | I | V |
|  | 43 | E | A |
|  | 125 | A | V |
|  | 203 | H | Q |
|  | 467 | T | A |
|  | 492 | Q | L |
| nsP3 | 175 | V | I |
|  | 331 | T | I |
|  | 371 | S | P |
|  | 392 | S | R |
|  | 396 | R | Q |
|  | 428 | S | N |
|  | 437 | A | E |
|  | 444 | G | R |
|  | 445 | A | V |
|  | 488 | T | A |
|  | 499 | H | Y |
|  | 511 | L | P |
|  | 550 | N | S |
|  | 563 | * | R |
| nsP4 | 75 | M | T |
|  | 222 | A | V |
|  | 255 | T | M |
|  | 499 | V | E |
|  | 519 | A | S |
|  | 568 | R | K |
| E2 | 82 | S | G |
|  | 372 | V | A |
|  | 382 | A | V |
| E1 | 220 | T | I |
|  | 291 | I | T |
|  | 299 | V | M |
|  | 396 | I | T |
|  | 431 | A | V |

**Supplementary table 1** The 36 amino acid residues in the RRC013 genome that differed consistently from all other strains (ONNV SG650, DakAr234, UgMP30, IbH10964 and IbH12628) identified by Illumina sequencing of all strains followed by multiple alignment comparison of the non-structural and structural open reading frame independently. Asterisks referring to the opal stop codon.

| **Gene** | **Amino acid** | **RRC013** | **IbH10964** | **IbH12628** | **Other strains** |
| --- | --- | --- | --- | --- | --- |
| nsP1 | 76 | R | R | K | R |
|  | 233 | L | F | L | L |
|  | 386 | I | I | T | I or T |
| nsP2 | 464 | H | Y | H | H |
| E2 | 59 | D | A | D | D |
| E1 | 343 | N | N | D | N |

**Supplementary table 2** The 6 amino acid residues that differed between the IbH10964 and the IbH12628 genome compared to RRC013 and the other ONNV strains (ONNV SG650, DakAr234, and UgMP30). Differences identified by Illumina sequencing of all strains followed by multiple alignment comparison of the non-structural and structural open reading frame independently.
